# An improved hybrid multi-criteria/multidimensional model for strategic industrial location selection: Casablanca industrial zones as a case study

**DOI:** 10.1186/s40064-015-1404-x

**Published:** 2015-10-20

**Authors:** Omar Boutkhoum, Mohamed Hanine, Tarik Agouti, Abdessadek Tikniouine

**Affiliations:** Laboratory of Information Systems Engineering, Department of Computer Science, Faculty of the Sciences Semlalia, Cadi Ayyad University, Marrakesh, Morocco

**Keywords:** Industrial location selection, Decision support system, Multi-criteria analysis, OLAP analysis

## Abstract

In this paper, we examine the issue of strategic industrial location selection in uncertain decision making environments for implanting new industrial corporation. In fact, the industrial location issue is typically considered as a crucial factor in business research field which is related to many calculations about natural resources, distributors, suppliers, customers, and most other things. Based on the integration of environmental, economic and social decisive elements of sustainable development, this paper presents a hybrid decision making model combining fuzzy multi-criteria analysis with analytical capabilities that OLAP systems can provide for successful and optimal industrial location selection. The proposed model mainly consists in three stages. In the first stage, a decision-making committee has been established to identify the evaluation criteria impacting the location selection process. In the second stage, we develop fuzzy AHP software based on the extent analysis method to assign the importance weights to the selected criteria, which allows us to model the linguistic vagueness, ambiguity, and incomplete knowledge. In the last stage, OLAP analysis integrated with multi-criteria analysis employs these weighted criteria as inputs to evaluate, rank and select the strategic industrial location for implanting new business corporation in the region of Casablanca, Morocco. Finally, a sensitivity analysis is performed to evaluate the impact of criteria weights and the preferences given by decision makers on the final rankings of strategic industrial locations.

**Electronic supplementary material:**

The online version of this article (doi:10.1186/s40064-015-1404-x) contains supplementary material, which is available to authorized users.

## Background

Strategic industrial location decisions have garnered considerable attention from the academic and business communities. Increasingly, it has been proved to be vital (Kapoor et al. 2008) especially for industrial and urban projects, which can accelerate the rate of economic growth, increase economic efficiency, minimize unnecessary cost, maximize the use of resources, improve investment climate, and promote the development of the regional economy (Yong 2005; Rao et al 2015). The science of “location selection” is truly multidisciplinary and representing a variety of scientific fields, ranging from business to operations research to computer science (Church and Murray 2008). In fact, the success or failure of most industrial businesses often depends on the formal business plan of these businesses, and also on the owner’s ability to choose his location within or among several industrial areas. In this context, a priori selection of a suitable industrial location is a complex process which involves a number of different potential criteria, such as cost of investment, availability of acquisition material, human resources, etc., that must be considered in selecting a strategic industrial location (Yong 2005).

Following these considerations, several contributions have been dedicated to the location selection problem using different multi-criteria decision making methods such as fuzzy Delphi, fuzzy AHP, ANP (Analytic Network Process), TOPSIS (Technique for Order Preference by Similarity to Ideal Solution) and PROMETHEE (Preference Ranking Organization METHod for Enrichment Evaluations). For instance, Chou et al (2008) presented a fuzzy multi-criteria decision making model based on fuzzy AHP for international tourist hotel location selection. Guneri et al. (Guneri et al. 2009) applied fuzzy ANP method to identify and select a suitable location for a shipyard. Also, Hsu (2010) employed ANP methodology to select the appropriate location for an international business office center in China. Önüt et al. (2010) presented an integrated fuzzy multi-criteria decision making approach based on fuzzy AHP and fuzzy TOPSIS methods for the selection of a suitable shopping center location in Istanbul, Turkey. Li et al. (2011) used the axiomatic fuzzy set clustering approach and TOPSIS technique to select a logistic center location. Bottero and Ferretti (2011) ranked sites for the location of a waste incinerator plant in the Province of Torino in Italy using ANP method. Athawale et al. (2012) applied PROMETHEE II to solve real time facility location selection problems. Choudhary and Shankar (2012) proposed a framework based on an STEEP-fuzzy AHP-TOPSIS for the evaluation and selection of thermal power plant location taking India as a case study. Furfaro et al (2012) presented the development of a model of an evolutionary fuzzy cognitive map model to select a landing site for scientific discoveries forced by the soft landing requirement in an area with safe lands. Ishizaka et al. (2013) used the weighted sum method, TOPSIS, and PROMETHEE for casinos location selection in the Greater London region. Kabir and Sumi (2014) used fuzzy AHP and PROMETHEE for power substation location selection, taking Bangladesh as a case study. Yunna and Geng (2014) provided a multi-criteria decision making framework based on AHP for the selection of solar–wind hybrid power station location in China. Moreover, Chang et al. (2015) have combined the fuzzy Delphi method, ANP, and TOPSIS to effectively make better decisions for optimal location selection in Taiwanese service apartments. Rao et al. (2015) presents a fuzzy multi-attribute group decision making technique based on a linguistic 2-tuple for the location selection of a City Logistics Centers from a sustainability perspective.

On the other hand, Geographic Information System (GIS) is also applied in many location selections due to its spatial capabilities to facilitate the geographical localization selection. Consequently, several GIS applications have been conducted in order to select the optimal location such as the contribution of Demesouka et al. (2013) combining GIS with AHP and TOPSIS to evaluate and select the appropriate location for landfill waste in the North of Greece. Rikalovic et al. (2014) propose GIS based multi-criteria analysis for industrial site selection in the region of Vojvodina, Serbia. Further, Latinopoulos and Kechagia (2015) proposed a GIS-based multi-criteria evaluation for wind farm site selection in Greece. Yang et al. (2015) present a new approach based on a combination of a set of machine learning algorithms and web GIS to evaluate potential sites for proposed hotel properties.

As a result, many methodologies, approaches, frameworks and applications have been proposed for the location selection problem (Önüt et al. 2010; Li et al. 2011; Choudhary and Shankar 2012; Demesouka et al. 2013; Kabir and Sumi 2014; Chang et al. 2015…). Most of them have focused firstly on classical and traditional criteria, such as reducing economic costs and maximizing customer service levels, instead of focusing on criteria evolving over time like those of sustainable development incorporating economic, social and environmental factors and on multidimensional data. In this context, to the best of our knowledge, very few of these contributions have emphasized their attention on the strategic industrial location selection, especially for its application in the Moroccan regions by using an integrated approach combining fuzzy multi-criteria analysis and OLAP analysis. In addition, it is difficult to clearly express the significance and character of criteria using traditional methods. Hence, combining the concept of fuzzy set theory and natural language with AHP method to evaluate the location selection criteria in a fuzzy environment is more convenient, allowing decision makers to adequately and freely express their ideas. These reasons have motivated us to propose an improved hybrid multi-criteria/multidimensional approach to select the strategic industrial location for the implantation of new business corporation in the region of Casablanca. The proposed approach has three essential stages. In the first stage, a decision making committee is formed in order to identify and select criteria for the alternative assessment phase. In the second stage, the importance weight is assigned to the selected criteria using integrated fuzzy AHP software. Lastly, OLAP analysis combined with multi-criteria analysis employs these weighted criteria as inputs to evaluate and select the strategic industrial location for the implantation of new business corporation in the region of Casablanca. Finally, we conduct a sensitivity analysis to evaluate the impact of criteria weights and the preferences given by decision makers on the final rankings of strategic industrial location.

This paper is organized as follows. In “Methodology”, we discuss our research methodology and develop our proposed hybrid approach. “Results and discussion” presents an empirical study illustrating the effectiveness and performance of our integrated approach. Finally, “Conclusion” contains some concluding remarks.

## Methodology

During this section, we discuss the various steps and tools constructing our proposed methodology, starting from evaluation of the selected criteria, assessment of potential alternatives and finally, presentation of the final results.

### Fuzzy AHP

The Analytic hierarchy Process (AHP), initially introduced by Saaty (1980), has becomes a powerful and flexible methodology in solving complex decision problems. In fact, the AHP process consists in representing a decision problem by a hierarchical structure reflecting the interactions between the various elements of the problem, then using pair-wise comparison judgments to identify and estimate the relative importance of criteria and alternatives. However, the AHP method has some shortcomings (Yang and Chen, 2004) due to its ineffectiveness when applied to an ambiguous problem with a high uncertainty. Therefore, several researchers, including those in “Background”, introduce fuzzy logic into the pairwise comparison of the AHP to compensate and deal with this type of fuzzy decision problem.

One of the latest FAHP methodologies is based on Chang’s extent analysis. It is relatively easier compared to many other approaches of FAHP. Hence, in this paper we prefer to utilize Chang (1996) extent analysis method to evaluate the importance weight of each selected criteria. The theoretical fundamentals of Chang’s extent analysis on FAHP were defined as follows (Gumus 2009):

Let ${\text{X}} = \left\{ {{\text{x}}_{ 1} ,{\text{ x}}_{ 2} , \ldots ,{\text{x}}_{\text{n}} } \right\}$ be an object set, and ${\text{G }} = \, \left\{ {{\text{u}}_{ 1} ,{\text{u}}_{ 2} ,{\text{u}}_{ 3} , \ldots ,{\text{u}}_{\text{m}} } \right\}$ as a goal set. According to the principles of Chang’s extent analysis, each object is considered and extent analysis for each of the goal, g_i_ is performed respectively. It means that *m* extent analysis values for each object can be obtained using the following signs:

1 $$M_{{g_{i} }}^{1} ,M_{{g_{i} }}^{2} , \ldots ,M_{{g_{i} }}^{m} \quad {\text{i }} = { 1},{ 2}, \ldots ,{\text{ n}},$$

where $M_{{g_{i} }}^{j} \left( {{\text{j}} = 1,{ 2}, \ldots ,{\text{ m}}} \right)$ are triangular fuzzy numbers. The followed steps of Chang’s extent analysis can be examined as explained below:

*Step 1* The value of fuzzy synthetic extent with respect to the ith object is defined as :

2 $$S_{i} = \sum\limits_{j = 1}^{m} {M_{{g_{i} }}^{j} } \otimes \left[ {\sum\limits_{i = 1}^{n} {\sum\limits_{j = 1}^{m} {M_{{g_{i} }}^{j} } } } \right]^{ - 1} .$$

The fuzzy addition operation of m extent analysis values must be performed for particular matrix to obtain $\sum\nolimits_{j = 1}^{m} {M_{{g_{i} }}^{j} }$ such that:

3 $$\sum\limits_{j = 1}^{m} {M_{{g_{i} }}^{j} } = \left( {\sum\limits_{j = 1}^{m} {l_{j} ,\sum\limits_{j = 1}^{m} {m_{j} ,} \sum\limits_{j = 1}^{m} {u_{j} } } } \right).$$

Then, we perform the fuzzy edition operation of m extent analysis values for a particular matrix to obtain $\left[ {\sum\nolimits_{i = 1}^{n} {\sum\nolimits_{j = 1}^{m} {M_{{g_{i} }}^{j} } } } \right]^{ - 1}$, such that:

4 $$\sum\limits_{i = 1}^{n} {\sum\limits_{j = 1}^{m} {M_{{g_{i} }}^{j} } } = \left( {\sum\limits_{i = 1}^{n} {l_{i} ,\sum\limits_{i = 1}^{n} {m_{i} ,} \sum\limits_{i = 1}^{n} {u_{i} } } } \right).$$

And the inverse of the vector in Eq. (4) is computed such that

5 $$\left[ {\sum\limits_{i = 1}^{n} {\sum\limits_{j = 1}^{m} {M_{{g_{i} }}^{j} } } } \right]^{ - 1} = \left( {\frac{1}{{\sum\nolimits_{i = 1}^{n} {u_{i} } }},\frac{1}{{\sum\nolimits_{i = 1}^{n} {m_{i} } }},\frac{1}{{\sum\nolimits_{i = 1}^{n} {l_{i} } }}} \right).$$

*Step 2* The degree of possibility of ${\text{M}}_{ 2} = \, \left( {{\text{l}}_{ 2} ,{\text{m}}_{ 2} ,{\text{u}}_{ 2} } \right) \, \ge {\text{ M}}_{ 1} = \, \left( {{\text{l}}_{ 1} ,{\text{m}}_{ 1} ,{\text{u}}_{ 1} } \right)$ is defined as

6 $$V(M_{2} \ge M_{1} ) = \sup \left[ {\hbox{min} (\mu_{{M_{1} }} (x),\mu_{{M_{2} }} (y))} \right]_{y \ge x}$$

and it can be represented as follows:

$$V(M_{2} \ge M_{1} ) = hgt(M_{1} \cap M_{2} ) = \mu_{{M_{2} }} (d)$$

7 $$\left\{ {\begin{array}{*{20}c} 1 & {if} & {m_{2} \ge m_{1} } \\ 0 & {if} & {l_{1} \ge u_{2} } \\ {\frac{{l_{1} - u_{2} }}{{(m_{2} - u_{2} ) - (m_{1} - l_{1} )}}} & {} & {otherwise} \\ \end{array} } \right.$$

where d is the ordinate of the highest intersection point between $\mu_{{M_{1} }}$ and $\mu_{{M_{2} }}$.

To be able to compare M1 and M2 we need both the values of V(M_1_ ≥ M_2_) and V(M2 ≥ M1).

*Step 3* The degree possibility for a convex fuzzy number to be greater than *k* convex fuzzy numbers ${\text{M}}_{\text{i}} \left( {{\text{i }} = { 1}, 2, \ldots ,{\text{k}}} \right)$ can be defined by

$${\text{V}}\left( {{\text{M }} \ge {\text{ M}}_{ 1} ,{\text{ M}}_{ 2} , \ldots ,{\text{ M}}_{\text{k}} } \right) \, 
= {\text{ V}}\left[ {\left( {{\text{M }} \ge {\text{ M}}_{ 1} } \right){\text{ and V}}\left( {{\text{M }} \ge {\text{ M}}_{ 2} } \right){\text{ and }} \ldots {\text{ and M }} \ge {\text{ M}}_{\text{k}} } \right]$$

8 $$= {\text{ min V}}\left( {{\text{M }} \ge {\text{ M}}_{\text{i}} } \right), \quad {\text{ i}} = 1,{ 2}, \ldots ,{\text{ k}}.$$

Assume that,

9 $${\text{d}}^{{\prime }} \left( {{\text{A}}_{{\text{i}}} } \right){\text{ = minV}}\left( {{\text{S}}_{{\text{i}}} \ge {\text{ S}}_{{\text{k}}} } \right){\text{.}}$$

For ${\text{k }} = { 1},{ 2}, \ldots ,{\text{ n}};{\text{ k}} \ne {\text{ i}}$. Then the weight vector is given by

10 $${\text{W}}^{\prime } \, = \, \left( {{\text{d}}^{\prime } \left( {{\text{A}}_{ 1} } \right),{\text{ d}}^{\prime } \left( {{\text{A}}_{ 2} } \right), \ldots ,{\text{ d}}^{\prime } \left( {{\text{A}}_{\text{n}} } \right)} \right)^{\text{T}} ,$$

where ${\text{A}}_{\text{i}} \left( {{\text{i }} = { 1},{ 2}, \ldots ,{\text{ n}}} \right)$ are *n* elements.

*Step 4* Via normalization, the normalized weight vectors are

11 $${\text{W}}^{\text{t}} = \, \left( {{\text{d}}\left( {{\text{A}}_{ 1} } \right),{\text{ d}}\left( {{\text{A}}_{ 2} } \right), \ldots ,{\text{ d}}\left( {{\text{A}}_{\text{n}} } \right)} \right)^{\text{T}}$$

where W is a non fuzzy number.

### OLAP system

**Fig. 1:**
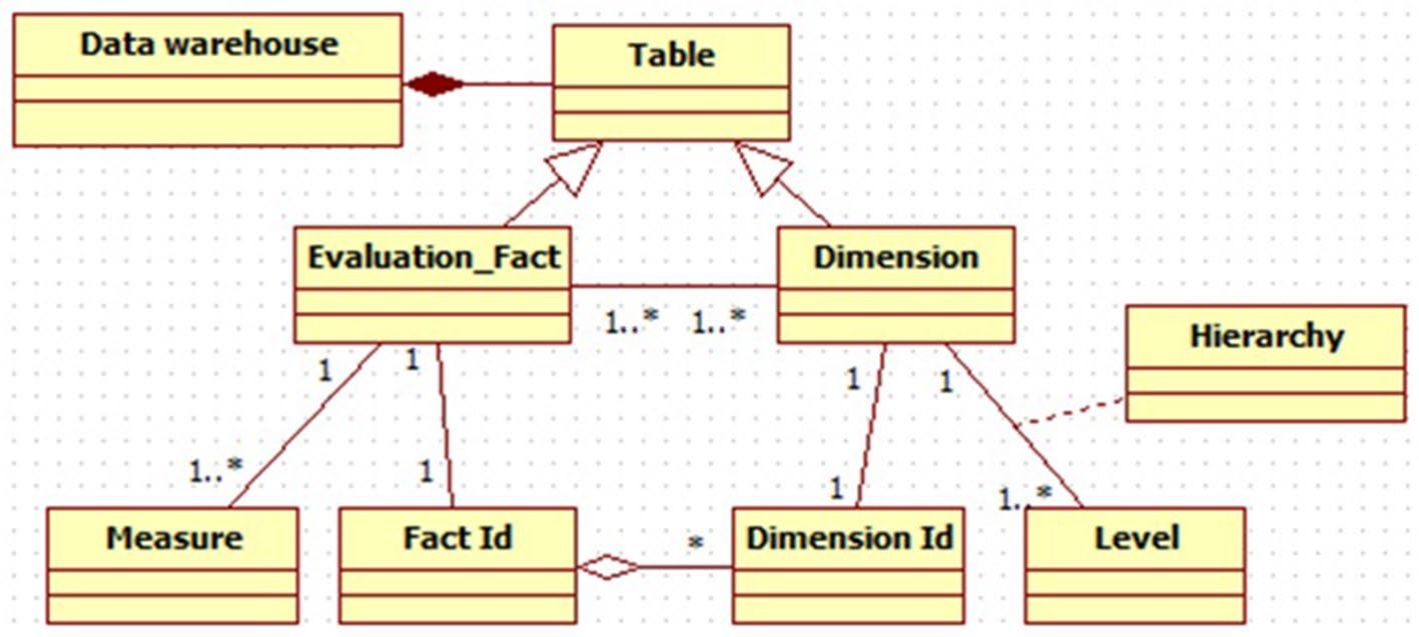
Data warehouse multidimensional modeling

Recently, decision support systems have been largely improved thanks to a large number of scientific researches. OLAP tools, being a decision making technology, appear as a complete system that provides helpful and necessary services for a rational and efficient treatment of intelligence data. In this kind of models, data are well organized multi-dimensionally so that the decision makers could analyze them interactively and iteratively at a detailed and/or aggregated level. The multidimensional structure (Kimball 1996) can be represented by a cube. A cube is composed of elements called cells. The cells contain the values of a fact, usually called measures. The cube axes correspond to the dimensions and they are graduated by members. In this multidimensional structure, the dimensions are hierarchies and therefore comprise a set of levels related by classification relationships (Fig. 1).

**Fig. 2:**
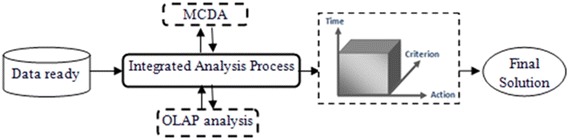
Abstract representation cycle of the OLAP-MCDA cube

The advantages of OLAP system are very numerous, however, this system has some shortcomings especially when we deal with more complex situations where several criteria (quantitative and/or qualitative) should be taken into account, which will certainly lead to bad consequences such as failure in achieving decision quality improvement, occasionally long analysis cycle times, and low decision makers’ satisfaction. In this context, combining multi-criteria decision making analysis (MCDA) with fuzzy set theory to enhance the analytical capabilities within OLAP system can offer an effective approach to resolve complex decision making problems. Hence, it is useful to envisage an optimized data model for OLAP cubes, taking into consideration various criteria on which we can apply new methods of MCDA as explained in Fig. 2.

The conceptual model used for this integrated approach is based on a star dimensional structure, which provides a fact table (OLAP-MCDA cube) as evaluation table that contains observable, measurable and digital data (Kimball and Ross 2002) circled by dimensions including the specific needs of decision makers as mentioned below:

*Action dimension* represents all actions, alternatives or solutions to be evaluated.

*Criteria dimension* includes criteria selected by the decision makers when defining objectives. They point the judgment on which an action is evaluated and measured.

*Time dimension* checks the impact of each criterion with respect to each action for a definite period of time.

**Fig. 3:**
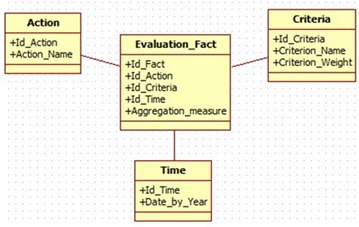
Multidimensional star schema

The proposed conceptual model used to construct our new OLAP cube is shown in Fig. 3.

The weighted sum function will be used as a multi-criteria analysis method due to its simplicity to be easily integrated within the XML file containing the OLAP cube. Hence, the aggregation of the criteria dimension values will be achieved by introducing different weighting in the evaluation process using this formula:

12 $$u\left( {a_{i} } \right) \, = \sum\limits_{j = 1}^{k} {v_{j} \cdot r_{ij} }$$

where u(a_i_) is the utility evaluated of ith alternative, v_j_ is the weight of jth criterion, r_ij_ is the utility evaluated of ith alternative for jth criterion

### The followed methodology

**Fig. 4:**
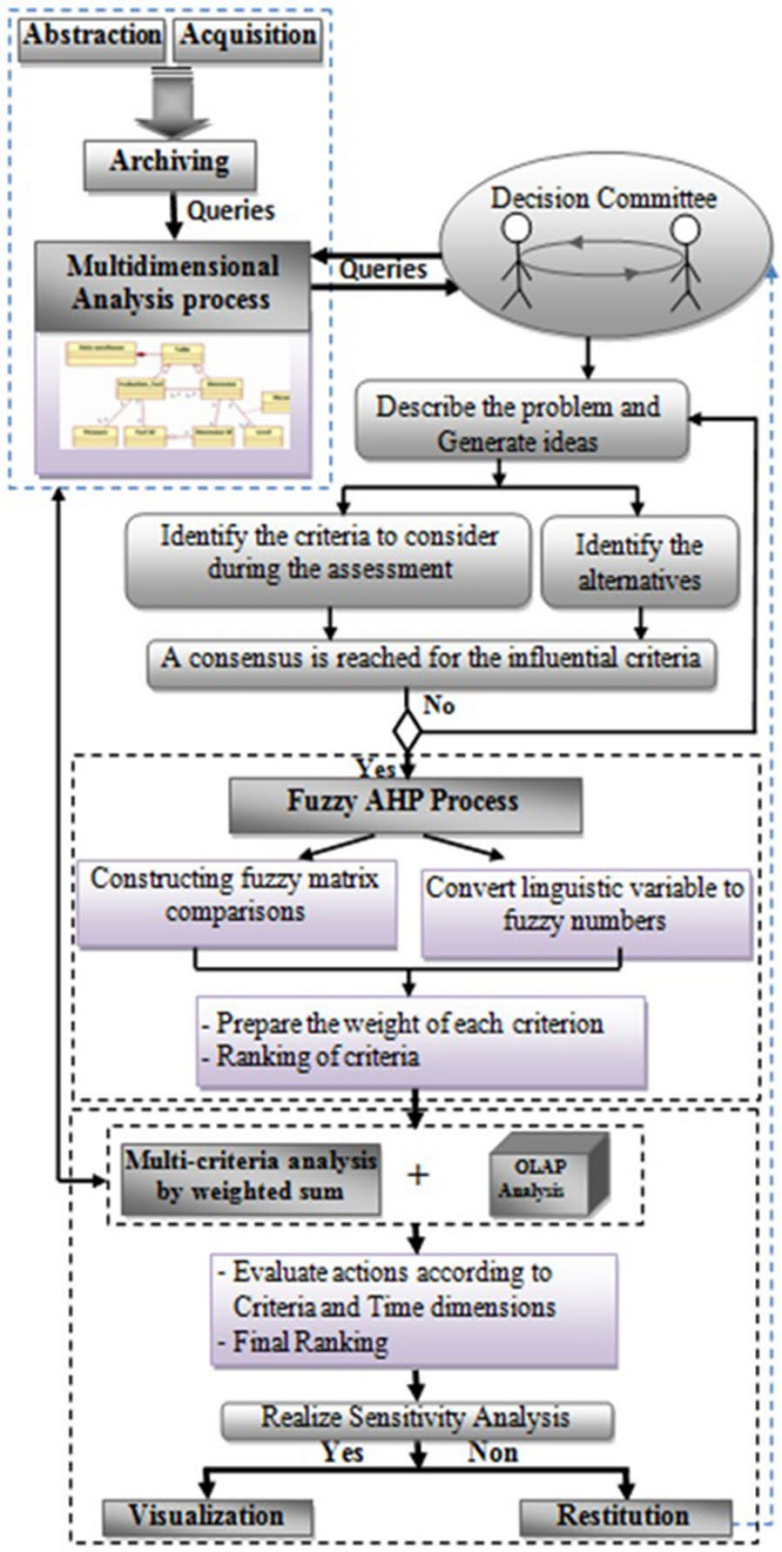
Proposed approach

The proposed hybrid multi-criteria/multidimensional model for the selection of strategic industrial location has three major processes as explained in Fig. 4:

*Process I* During this process, a decision making committee is formed of two experts, one project manager and one real estate consultant in order to determine the most influential criteria required to evaluate the proposed strategic industrial location. The committee begins by a detailed description of the problem and generates ideas about the needed criteria to be implemented when making the decision. It is ended when a consensus is reached for the selected criteria.

*Process II* After a consensus is reached for the identified criteria, the committee occurs at the fuzzy AHP process to construct the pairwise comparison matrices, and converts the linguistic appreciations of decision makers assigned to each criterion using Table 1 to easily derive corresponding values of fuzzy numbers, and finally calculates the importance weights of each criterion.

**Table 1:** Fuzzy comparison measures (Gumus 2009)

Linguistic terms	Triangular fuzzy numbers
Very good (VG)	(7, 9, 9)
Good (Gd)	(5, 7, 9)
Preferable (P)	(3, 5, 7)
Weak advantage (WA)	(1, 3, 5)
Equal (EQ)	(1, 1, 1)
Less WA	(1/5, 1/3, 1)
Less P	(1/7, 1/5, 1/3)
Less G	(1/9, 1/7, 1/5)
Less VG	(1/9, 1/9, 1/7)

*Process III* The main objective of this process is to evaluate and select the strategic industrial location using OLAP optimized data model. This new model combines the analytical capabilities of OLAP system with the weighted sum as a multi-criteria analysis method characterized by its mathematical accessibility over the other MCDA methods. The relative importance/weights of the evaluation criteria obtained from the fuzzy AHP process are taken into account as inputs in this process which will enable us to identify the candidate alternative as a final result.

### Computational study

In this section, we show the numerical experiments for the strategic industrial location selection using our hybrid multi-criteria/multidimensional approach.

### Problem description

**Fig. 5:**
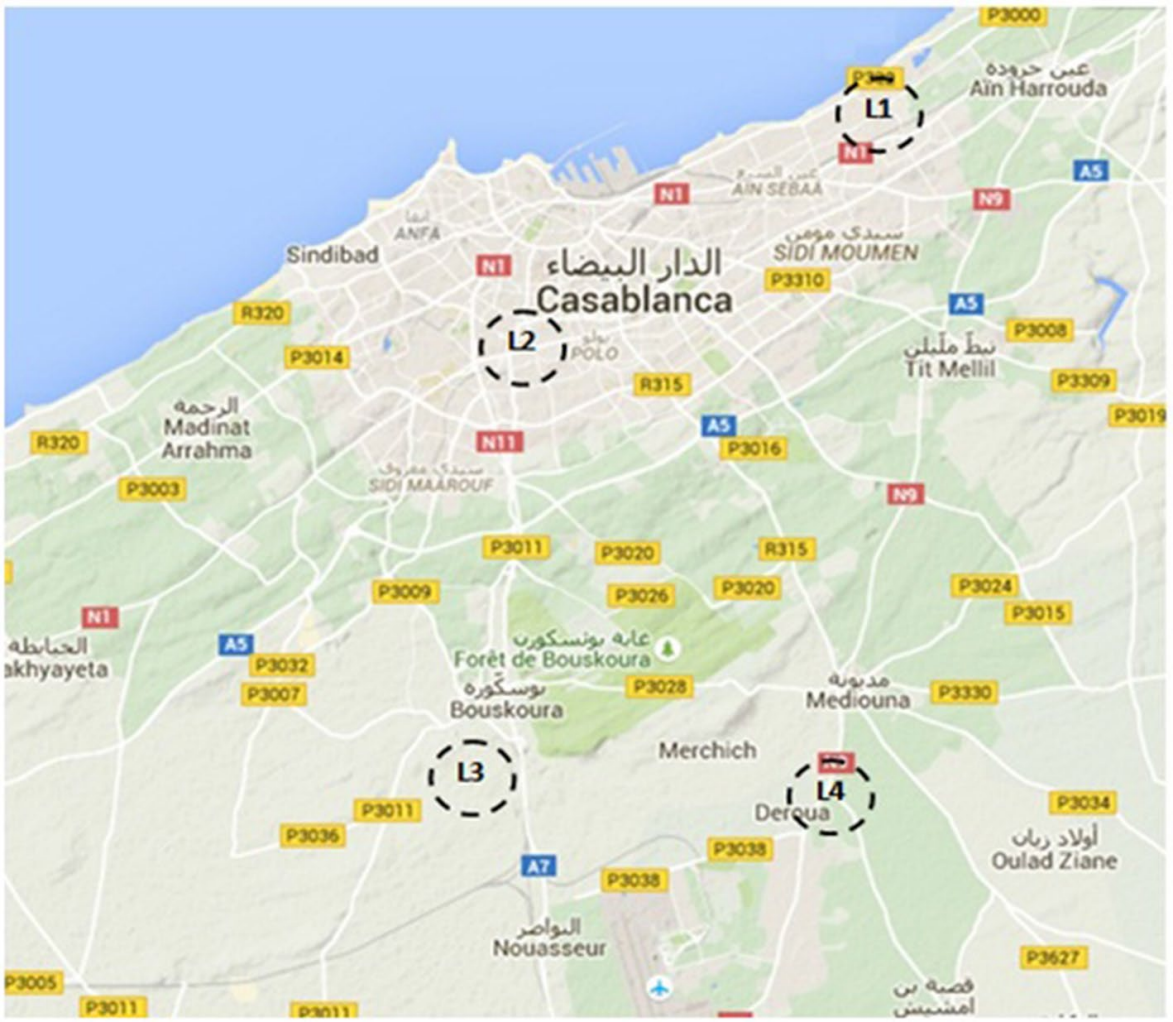
Alternative large industrial locations in Casablanca

According to the Moroccan Department of Statistics, Casablanca, as the largest and the most populated agglomeration in the Maghreb, accounts for more than 50 % of the total capital investment, and provides over 48 % of the industrial employment for many years. Also, Casablanca has more than six large industrial locations, of which the decision making committee has selected the four most active locations (L1, L2, L3 and L4) with respect to their competitive importance (Fig. 5).

In this context, choosing the appropriate industrial zone to install an industrial company in the region of Casablanca involves making a full implanting study to achieve the right choice, and thus find the right location, that is often strategic for the development of the company. This corporation of central industrial equipment superstructure (automobile industry) composed of a production chain that produces 10 units per day, and aims to target the Moroccan and African markets.

**Fig. 6:**
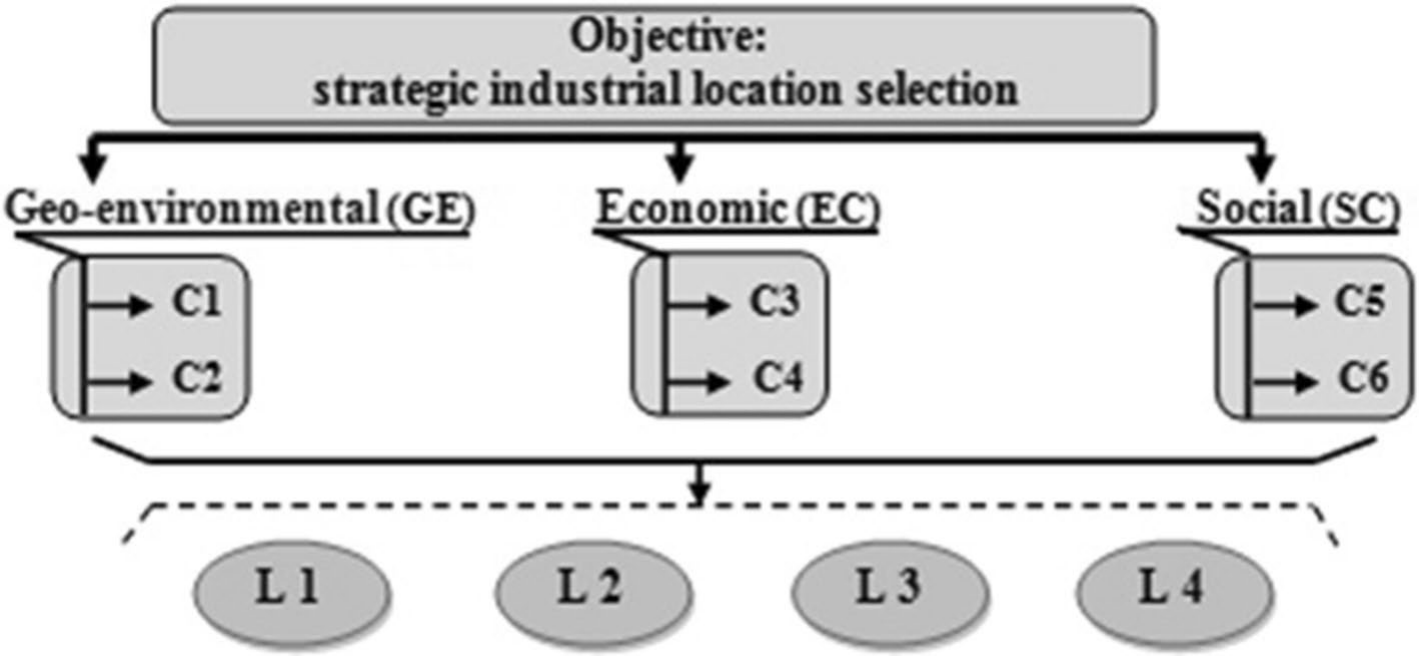
Hierarchical structure used for strategic industrial locations selection

As illustrated in Fig. 6, the hierarchical structure of this decision-making problem consists of four levels: The objective is shown in the highest level. A set of criteria to take into consideration when evaluating large industrial locations is identified by a decision making committee, exploring available literature. Thus, the committee reached to select three sustainability criteria evolving over time which are Geo-environmental, Economic and Social in the second level, while six sub-criteria (limited to the most influencing sub-criteria: C1, …, C6) are classified on the third level . The last level of hierarchy includes four large industrial locations (L1, …, L4).

The selected criteria arising from a sustainability perspective to evaluate and select the potential industrial location (Choudhary and Shankar 2012; Mohammadi et al. 2014; Chen et al. 2014; Rao et al. 2015) are as follows:

#### Geo-environmental criteria (GE)

*Situation and proximity of green areas(C1)* The implantation site should be at the center or can be located on the periphery or outside a city or urban area. The proximity of green areas, a low business tax and funding programs for business creators are all factors facilitating the start-up companies.

*Land (C2)* The size and quality of future commercial premises are influenced by the price of land, the collection rate of business tax, the prices of energy and the legal provisions on noise pollution, and possibility to expand the business.

### Economic criteria (EC)

*Cost (C3)* This criterion takes into consideration the total cost of the acquisition, rents and charges (eg. for equipment, connection, administrative costs) and waste disposal costs…

*Competition and provision (C4)* The role of local competition in the commercial project, and ease and flexibility of the supply of raw materials, commodities, consumer articles and energy are to be taken into account during the evaluation process.

#### Social criteria (SC)

*Close proximity to the customer base and traffic lanes (C5)* For this criterion, we discuss the access flexibility that customers, suppliers and employees can smoothly have, and the proximity of an airport, a train station or highway in regard to the corporation.

*Manpower and information exchange (C6)* This implies the attractiveness and motivation of skilled staff of the company, as well as the cultural offer and the attractiveness of the location in relation to the free time. The implication of private and public consulting institutions regarding the exchange of information is also to be mentioned. In addition to the presence of higher education institutions with which it is possible to exchange information and develop other forms of cooperation.

## Results and discussion

In the following stage, the weights of criteria and sub-criteria are calculated using fuzzy-AHP, and these calculated weight values are used as input in the OLAP-MCDA process. Then, after OLAP-MCDA calculations, evaluation of the alternatives (strategic industrial location) and selection of the most suitable one is performed. At the conclusion, our results are checked and analyzed in detail using sensitivity analysis.

### Fuzzy AHP process

During this process, we construct the pairwise comparisons of the main criteria and their sub-criteria using Table 1 for linguistic terms and TFN (Triangular Fuzzy Numbers) scale. Due to space limitation and the similarity of the other calculations for each comparison matrix, we only provide the evaluation matrices of three members of decision making committee (DM_1_, DM2 and DM_3_) as mentioned in Tables 2 and 3.

**Table 2:** Comparison matrix for the main criteria using linguistic variables

Objective	EC			GE			SC		
	DM_1_	DM_2_	DM_3_	DM_1_	DM_2_	DM_3_	DM_1_	DM_2_	DM_3_
EC	EQ	EQ	EQ	P	WA	WA	WA	P	EQ
GE	L.WA	L.WA	L.P	EQ	EQ	EQ	L.P	EQ	L.WA
SC	EQ	L.P	L.WA	WA	EQ	P	EQ	EQ	EQ

**Table 3:** The evaluation matrix for the main criteria using TFN scale

Objective	EC	GE	SC
EC	(1, 1, 1)	(1, 3.667, 7)	(1, 3, 7)
GE	(0.143, 0.273, 1)	(1, 1, 1)	(0.143, 0.511, 1)
SC	(0.143, 0.333, 1)	(1, 1.957, 7)	(1, 1, 1)

To simplify the calculation steps, we provide a spreadsheet of Microsoft Excel (see Fig. 15 in Appendix 1 and Additional file 1 for group decision matrix), and a Java application to manage individual appreciations of policy makers (see Fig. 16 in Appendix 2 treating the individual appreciation of DM2). We present in the following, an example of calculations of the weights for main criteria using Chang’s extent analysis approach.

The values of fuzzy synthetic extent (from Table 3) are evaluated as follows:

$$\begin{aligned} {\text{S}}_{\text{EC}}& = \, \left( { 3,{ 7}. 6 6 7,{ 13}} \right)*\left( { 6. 4 2 9,{ 12}. 7 4 1,{ 24}. 9 9 3} \right)^{ - 1} \hfill \\ &= \, \left( {0. 1 1 1, \, 0. 60 2,{ 2}. 3 3 3} \right) \hfill \\ \end{aligned}$$

$$\begin{aligned} {\text{S}}_{\text{GE}} &= \, \left( { 1. 2 8 6,{ 1}. 7 8 4,{ 3}} \right)* \, \left( { 6. 4 2 9,{ 12}. 7 4 1,{ 24}. 9 9 3} \right)^{ - 1} \hfill \\ & = \, \left( {0.0 4 8, \, 0. 1 40, \, 0. 4 6 7} \right) \hfill \\ \end{aligned}$$

$$\begin{aligned} {\text{S}}_{\text{SC}}& = \, \left( { 2. 1 4 3,{ 3}. 2 90,{ 9}.000} \right)*\left( { 6. 4 2 9,{ 12}. 7 4 1,{ 24}. 9 9 3} \right)^{ - 1} \hfill \\ & = \, \left( {0.0 7 9, \, 0. 2 5 8,{ 1}. 400} \right). \hfill \\ \end{aligned}$$

Then these vectors will be used to calculate V values as shown in Table 4.

**Table 4:** V values result

V(S_col_ ≥ S_row_)	Criteria		
	EC	GE	SC
Column ≥ row	–	0.435	0.790
Column ≥ row	1	–	1
Column ≥ row	1	0.766	–

Thus, the weight vector from Table 4 is calculated as W′= (1, 0.435, 0.790), and the normalized weight vector is obtained as W^t^ = (0.450, 0.196, 0.355)^T^

Following the same systematic approach for the other evaluations, we get the priority weights correspondingly as explained below:

For sub-criteria (C1, C2): W_EC_ = (0.697, 0.307)^T^

For sub-criteria (C3, C4): W_GE_ = (0.179, 0.801)^T^

For sub-criteria (C5, C6): W_SC_ = (0.132, 0.870)^T^

As a summary, we provide in Table 5 the final importance weight and final ranking of all evaluation criteria.

**Table 5:** Final criteria weight

Criterion/sub criterion	Local weight	Global weight	Rank
EC	0.450		
C1	0.697	0.314	1
C2	0.307	0.138	4
GE	0.196		
C3	0.179	0.035	6
C4	0.801	0.157	3
SC	0.355		
C5	0.132	0.047	5
C6	0.870	0.309	2

### OLAP-MCDA analysis process: evaluation and ranking of alternatives

As explained in the previous approach, the importance weight assigned to the selected criteria will be used as input in the OLAP-MCDA process to evaluate and select the most appropriate alternative.

The objective is to select the suitable large industrial zone in Casablanca for implanting new industrial corporation taking into account all selected criteria proposed above. The value of each criterion with respect to each location is controlled during the period 2000–2014.

**Fig. 7:**
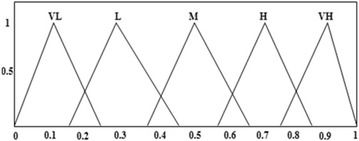
Linguistic scale for evaluation

The appreciations of decision makers for the evaluation of alternatives with respect to all specified criteria will be performed using linguistic scale for evaluation (Fig. 7; Table 6).

**Table 6:** Transformation for fuzzy membership functions

Linguistic	Membership function
Very low (VL)	(0.00, 0.10, 0.25)
Low (L)	(0.15, 0.30, 0.45)
Medium (M)	(0.35, 0.50, 0.65)
High (H)	(0.55, 0.70, 0.85)
Very High (VH)	(0.75, 0.90, 1.00)

**Fig. 8:**
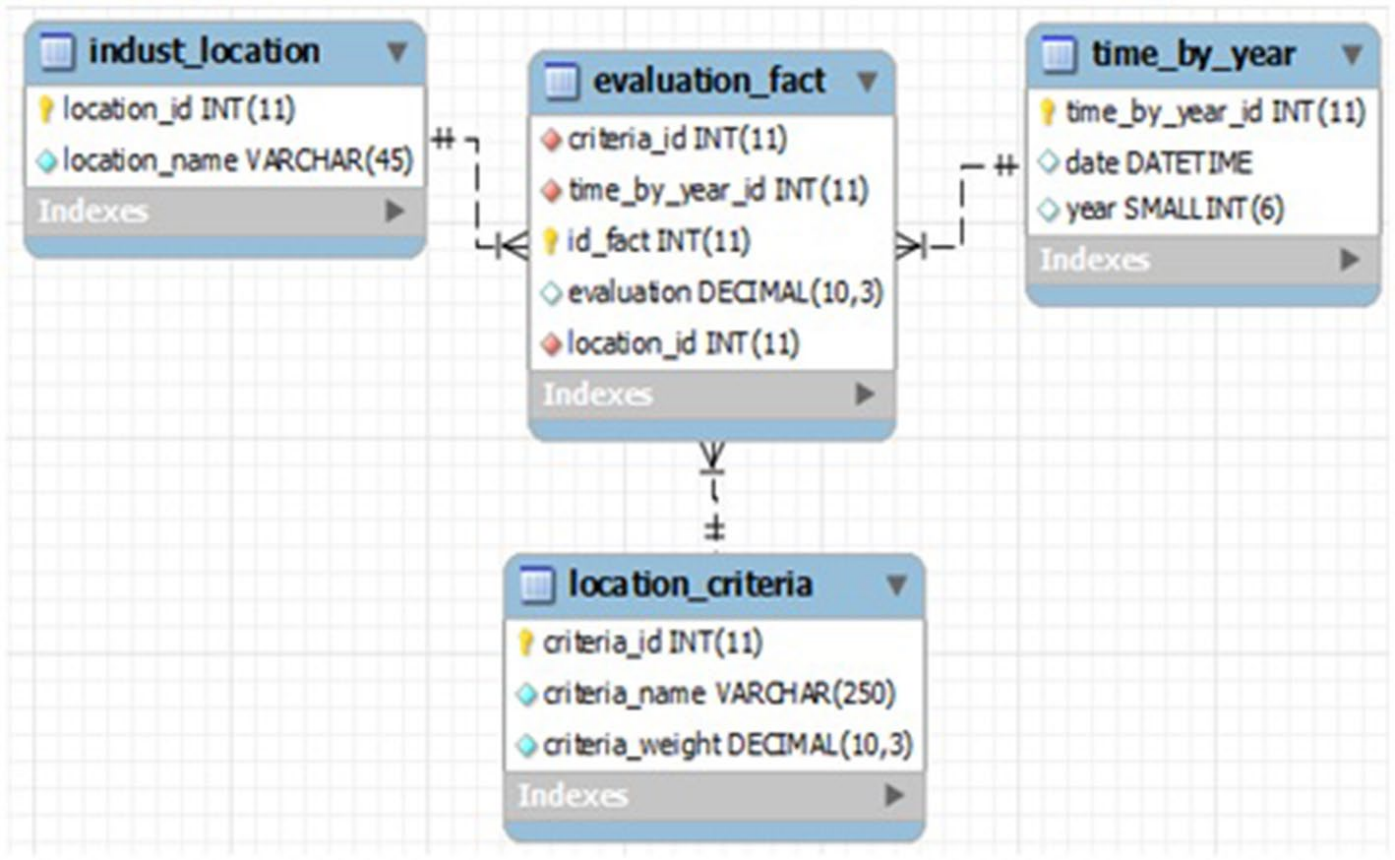
Star schema of OLAP-MCDA cube

We provide in Fig. 8, before any calculations, the analysis and modelling of the problem using a multidimensional star schema.

At this stage, we take into consideration the appreciations of decision makers for each criterion over a definite period of time (Table 7), and proceed to the evaluation of the four potential locations.

**Table 7:** The decision makers’ judgments over a defined period of time

Criteria	Time	L1	L2	L3	L4	Weight
C1	2000	L	M	L	VL	0.314
	2007	VL	H	L	H	
	2014	H	M	H	M	
C2	2000	VL	L	M	L	0.138
	2007	M	M	H	L	
	2014	M	M	H	M	
C3	2000	L	L	M	VL	0.035
	2007	M	M	M	L	
	2014	M	VH	H	M	
C4	2000	VL	M	L	L	0.157
	2007	L	M	M	L	
	2014	L	H	M	M	
C5	2000	M	L	VL	L	0.047
	2007	M	L	L	M	
	2014	H	M	M	VH	
C6	2000	L	L	H	M	0.309
	2007	M	L	H	H	
	2014	M	M	VH	H	

In the following steps, we use an open source OLAP server called Mondrian server (Pentaho community 2015), to bring multidimensional analysis and perform typical OLAP navigations like roll up, drill down, slice, dice, and pivot. To simplify this navigation, we used a JPivot interface, which is a JSP (JavaServer Pages) custom tag library. This will help us use MDX (Multidimensional Expressions) queries and XML language through this interface to screen very fast for a particular subset of the data from the XML file containing our OLAP-MCDA cube. The hybrid cube contains a measure called ‘evaluation’, and ‘weighted sum’, ‘multi-criteria aggregation’ as calculated members.

**Fig. 9:**
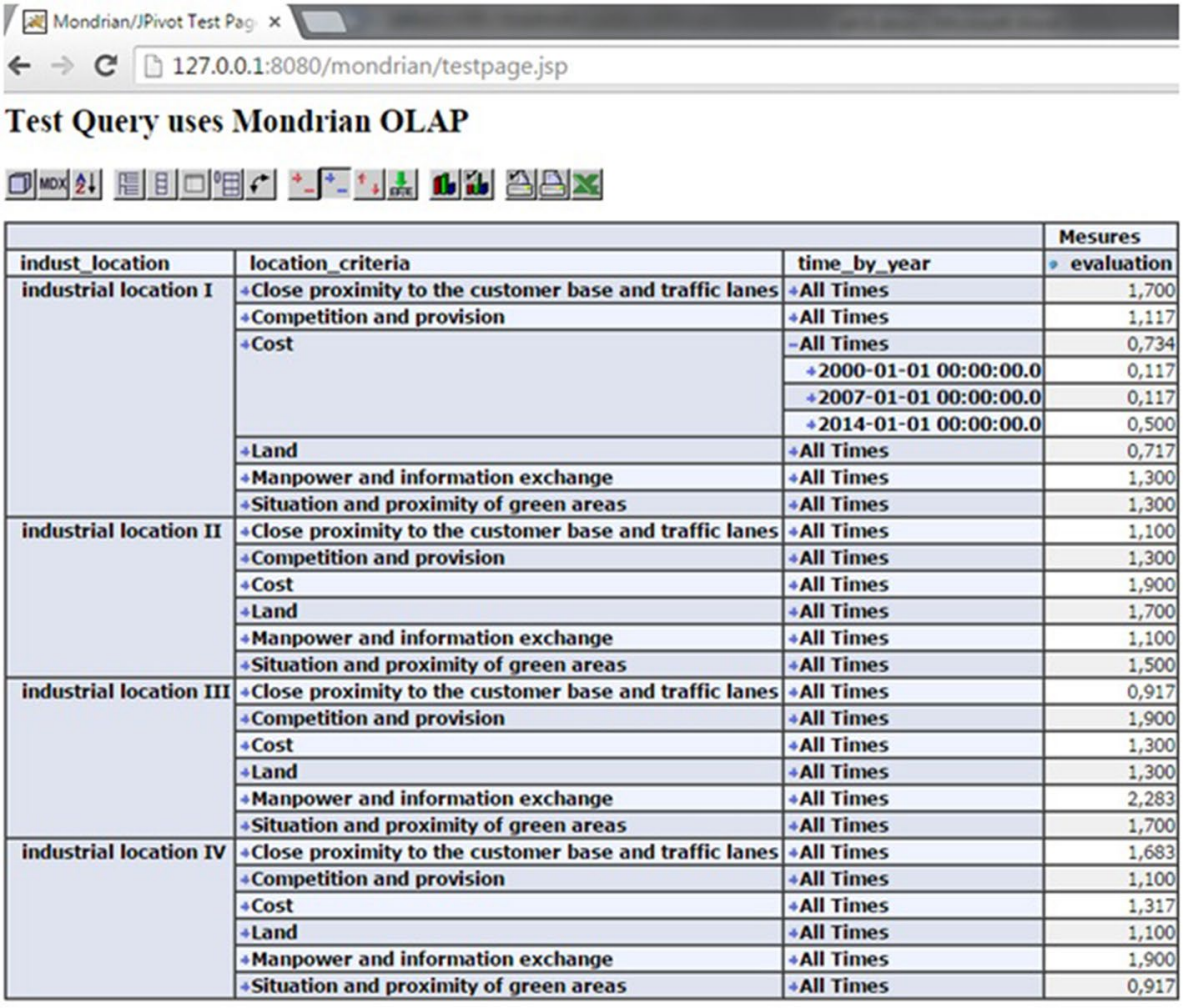
Hybrid cube representation using OLAP JPivot client of Mondrian server

**Fig. 10:**
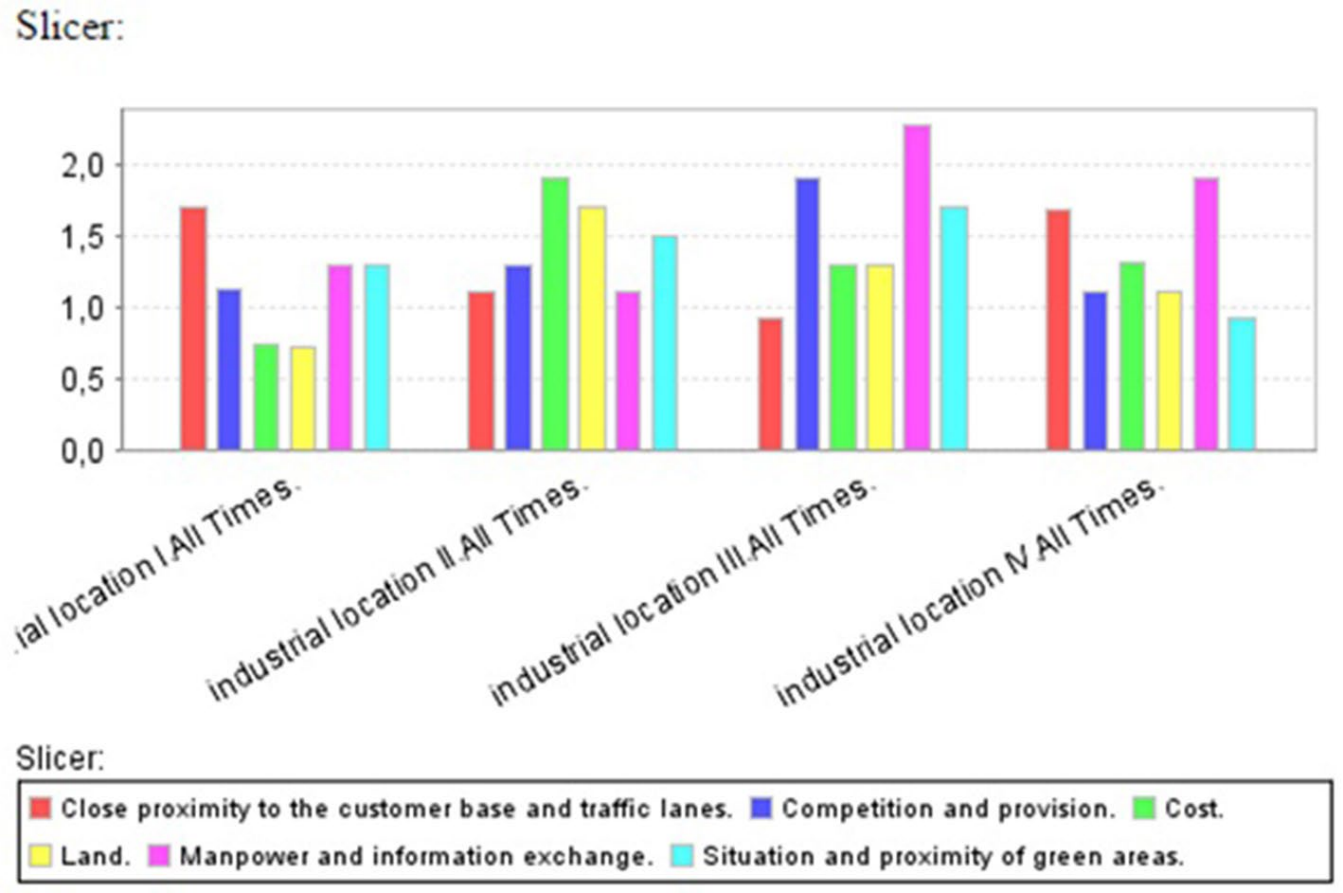
Criteria evaluation for each itinerary

The representation of our hybrid cube data is illustrated in Fig. 9 using MDX query:

By using MDX query, we can also illustrate the importance and impact of the selected criteria on each industrial location as mentioned in Fig. 10.

**Fig. 11:**
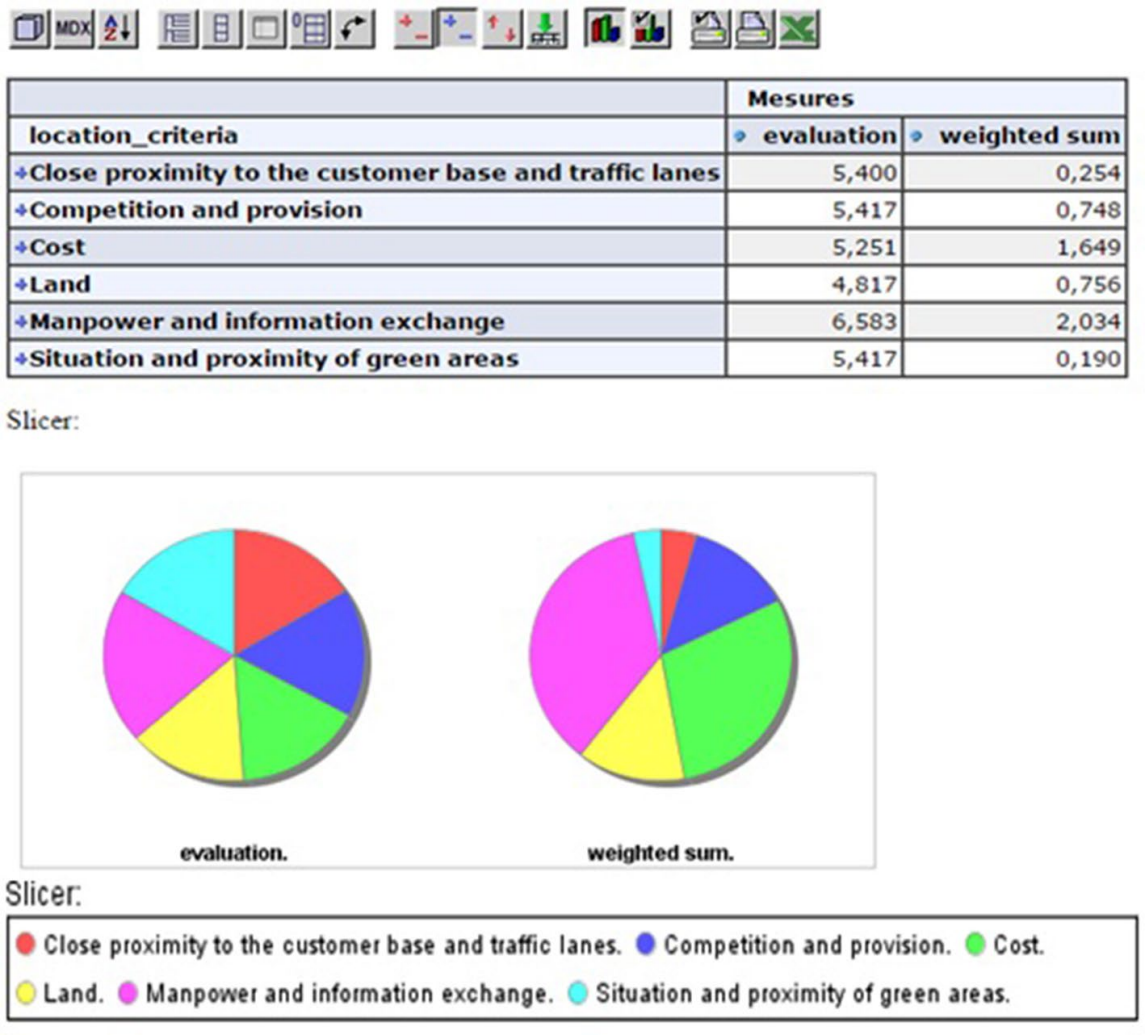
Effect and result of weighted sum on each criterion

Also, based on the decision maker’s judgments, we can verify the effect of the weighting on the importance of each criterion (Fig. 11) using Eq. (11).

**Fig. 12:**
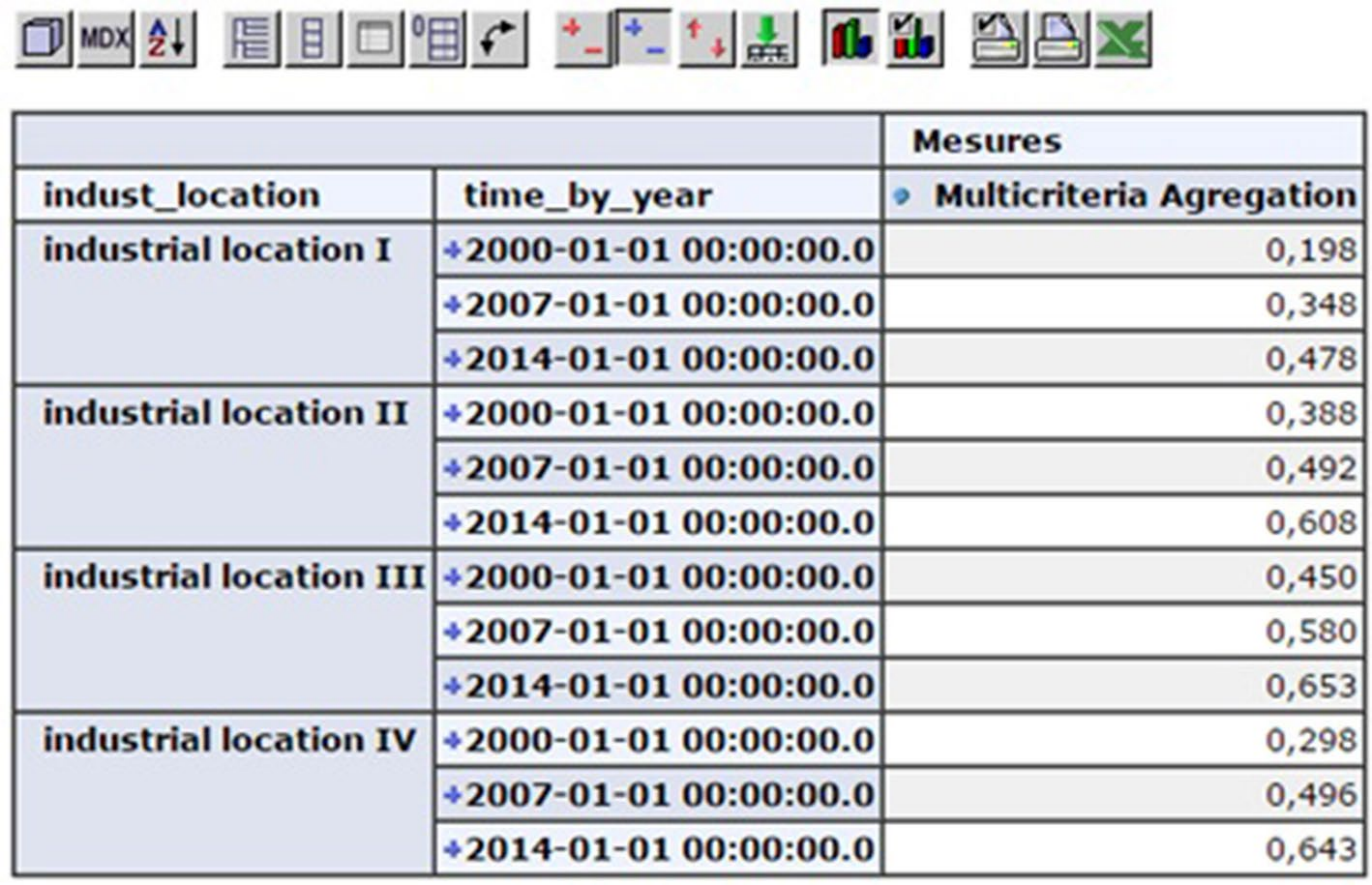
Aggregated evaluation per year for each industrial location

At this stage, we create and add a new calculated member “Multi-criteria Aggregation” to the criteria dimension to allow aggregation of the evaluation criteria according to the method of weighted sum, as shown in Fig. 12.

**Fig. 13:**
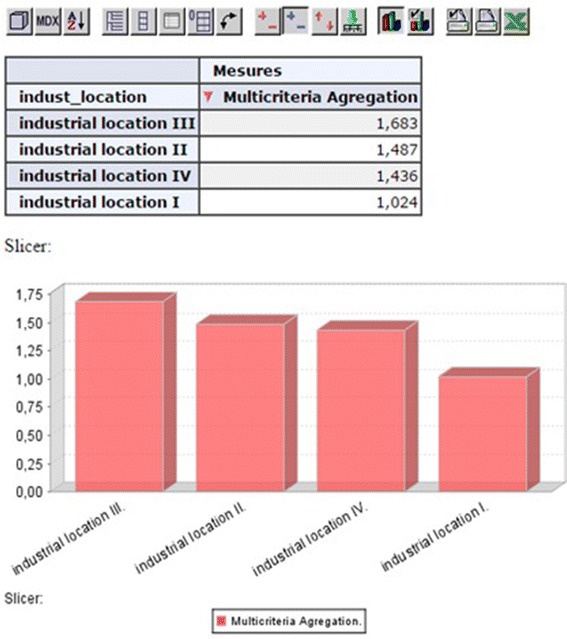
Final result

By exploiting the analytical mechanisms of OLAP server to move up in the hierarchy of the cube, the representation of the results are performed after the final ranking of multi-criteria aggregation for all locations as graphically shown in Fig. 13.

### Sensitivity analysis

As illustrated in Fig. 13, the final evaluation of potential industrial location is provided by using the visualization mechanisms of the OLAP Mondrian server. The most appropriate industrial location is the one with the highest score as mentioned graphically in Fig. 13, which revealed that industrial location III (L3) is the preferred location with a score of 1.683, followed by L2 (1.487), L4 (1.436) and finally L1 (1.024).

**Fig. 14:**
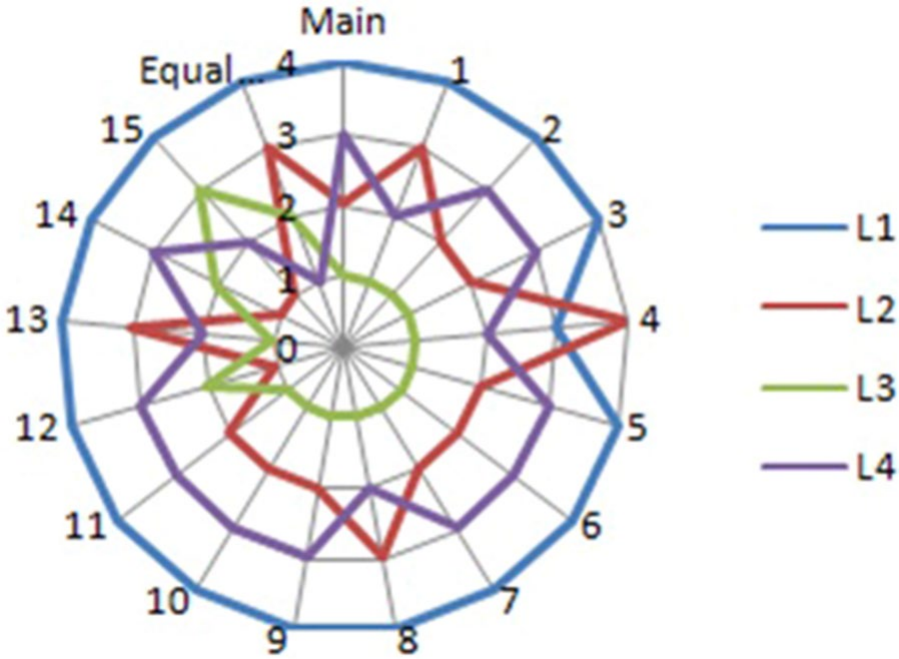
Final results of sensitivity analysis (multi-criteria aggregation scores)

**Fig. 15:**
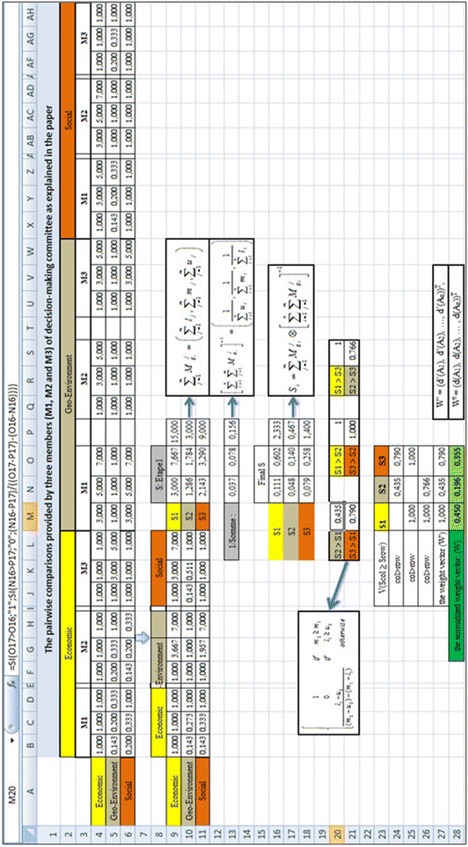
The computational steps of fuzzy AHP process

**Fig. 16:**
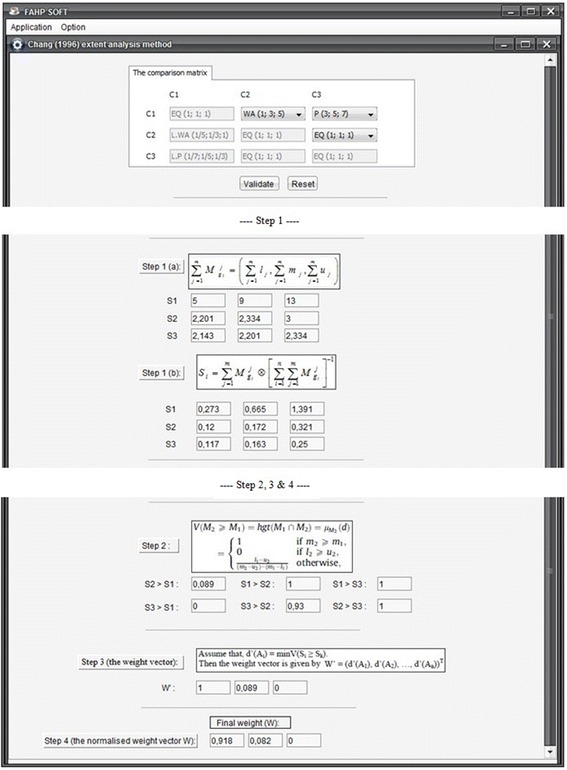
The proposed software prototype to simplify the process of calculating the criteria weight

With the aim of assessing the impact of decision makers’ risks to the final location ranking supplied previously, a sensitivity analysis which is presented in Table 8 is carried out. The main objective as suggested in many contributions such as (Mousavi et al. 2013; Zhu et al. 2015; Mosadeghi et al. 2015), is to check for the possible changes that may influence the final evaluation results listed in Fig. 13. Depending on this sensitivity analysis, exchanging each criterion’s weight with another and keeping practically the other weights the same, is performed gradually in fifteen combinations on which the original result is described as the main combination (see Additional file 2 for more detail). Also, the influence resulting from the performance of each combination on the final classification is examined, and the computational results are summarized in Table 8. Thus, the sensitivity results, as visualized in Fig. 14, show that the ‘L3’ remains the best location choice in nearly all combinations, except combinations 12, 14 and 15 on which the criterion C6 is exchanged respectively with C3, C4 and C5, more precisely, when the weight of C6 criterion is reduced to less than 30 %. L2 is classified as the second best industrial location in nine combinations by excluding combinations where C1 has lost more than 50 % of its weight (combination 1 and 4), and also when C2 and C4 criteria have reached the weight of 0.047. Similarly, L4 is ranked as the third best location in eleven combinations followed by L1 as the last choice in almost all combinations.

**Table 8:** Sensitivity analysis

Combinations	Criteria weights						Alternative rankings			
	C1	C2	C3	C4	C5	C6	L1	L2	L3	L4
Main	0.314	0.138	0.035	0.157	0.047	0.309	4	2	1	3
1	0.138	0.314	0.035	0.157	0.047	0.309	4	3	1	2
2	0.035	0.138	0.314	0.157	0.047	0.309	4	2	1	3
3	0.157	0.138	0.035	0.314	0.047	0.309	4	2	1	3
4	0.047	0.138	0.035	0.157	0.314	0.309	3	4	1	2
5	0.309	0.138	0.035	0.157	0.047	0.314	4	2	1	3
6	0.314	0.035	0.138	0.157	0.047	0.309	4	2	1	3
7	0.314	0.157	0.035	0.138	0.047	0.309	4	2	1	3
8	0.314	0.047	0.035	0.157	0.138	0.309	4	3	1	2
9	0.314	0.309	0.035	0.157	0.047	0.138	4	2	1	3
10	0.314	0.138	0.157	0.035	0.047	0.309	4	2	1	3
11	0.314	0.138	0.047	0.157	0.035	0.309	4	2	1	3
12	0.314	0.138	0.309	0.157	0.047	0.035	4	1	2	3
13	0.314	0.138	0.035	0.047	0.157	0.309	4	3	1	2
14	0.314	0.138	0.035	0.309	0.047	0.157	4	1	2	3
15	0.314	0.138	0.035	0.157	0.309	0.047	4	1	3	2
Equal weights	0.167	0.167	0.167	0.167	0.167	0.167	4	3	2	1

The sensitivity analysis results demonstrate that the ranking of the strategic industrial location has changed considerably on the equally weighted criteria, which explains that weight of criteria found consistently form an important step in our proposed hybrid model. As a result, the sensitivity analysis carried out indicates that the weights affect the ranking of alternatives, which will enable the decision committee to enhance its decision making process by fitting weighting and scoring, and performing sensitivity analysis.

## Conclusion

The purpose of this paper is to present an improved hybrid multi-criteria/multidimensional model based on fuzzy multi-criteria analysis and OLAP analysis for strategic industrial location selection in the region of Casablanca. The location selection is achieved by integrating the three dimensions of sustainability, namely, environmental, economic, and social. To solve the problem of selection criteria interdependency, a decision making committee is met to identify the evaluation criteria as the first stage. We only retain six important criteria to structure the hierarchy for selecting the optimal industrial location. We propose that forthcoming research studies integrate more criteria in order to make more precise estimates. In the second stage, fuzzy AHP is used to assign the importance weights to each criterion. These calculated weights, in the last stage, are employed in the OLAP analysis process as inputs to evaluate and select the strategic industrial location for implanting new business corporation in the region of Casablanca.

The application of our integrated methodology allows the policy makers of a company not only to determine the significant criteria, but also to compare, evaluate and select the potential alternatives appropriately, which can make better decisions in selecting the optimal industrial locations for implanting new business corporation. In this context, a sensitivity analysis is performed for the case study in order to better evaluate the risk of decision makers’ perception. The provided results are more objective and the imprecision is addressed and quantified properly.

Besides, different multi-criteria techniques such as PROMETHEE, TOPSIS and VIKOR can be employed in the location selection problem, as the contributions of (Taylana et al. 2014; Beikkhakhian et al. 2015; Chen 2015), and comparison of the results can be presented. The main difference between these techniques, implemented in many studies, and our hybrid analytical model consist at the ability to control the temporal evolution (time dimension’s role) of a given problem by taking advantage of the analytical and technical flexibilities that OLAP systems can provide.

## Appendix 1

See Fig. 15.

## Appendix 2

See Fig. 16.

## Additional files

10.1186/s40064-015-1404-x The computational steps of fuzzy AHP process.
10.1186/s40064-015-1404-x Final results of sensitivity analysis (Multicriteria aggregation scores) for each combination as mentioned in Table 8.
