# Supplementary material for: An improved hybrid multi-criteria/multidimensional model for strategic industrial location selection: Casablanca industrial zones as a case study
Source: Springerplus. 2015 Oct 20;4:628. doi: 10.1186/s40064-015-1404-x (PMC4631319; doi:10.1186/s40064-015-1404-x)

**An improved hybrid multi-criteria/multidimensional model for strategic industrial location selection: Casablanca industrial zones as a case study**

**Final results of sensitivity analysis (Multi-criteria aggregation scores) for each combination as mentioned in Table 8.**

Combination 1


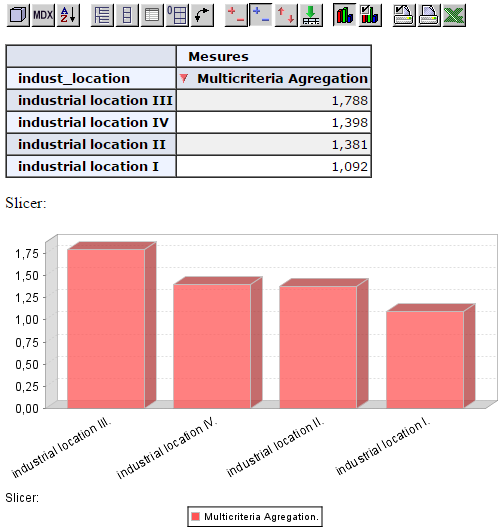


Combination 2


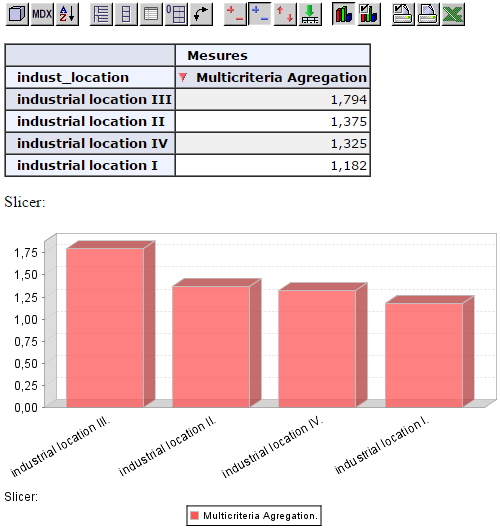


Combination 3


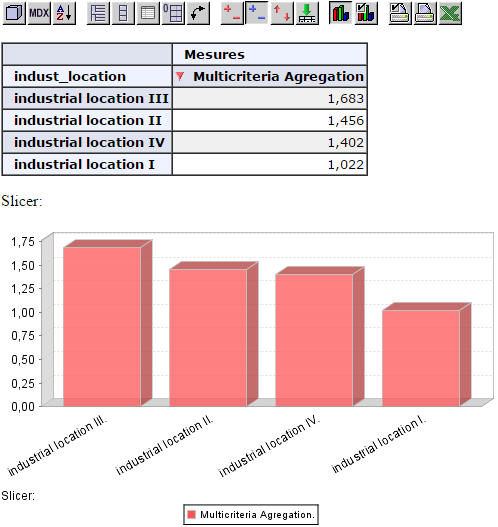


Combination 4


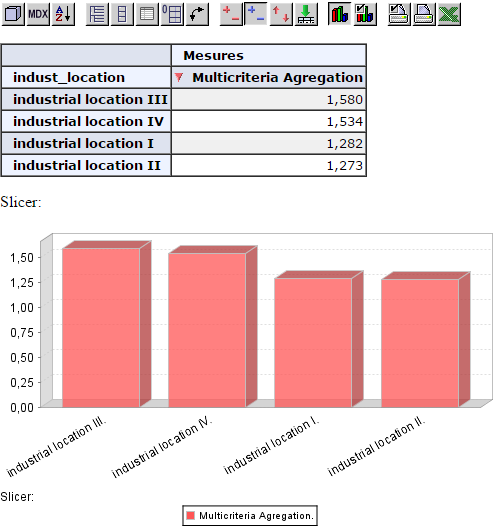


Combination 5


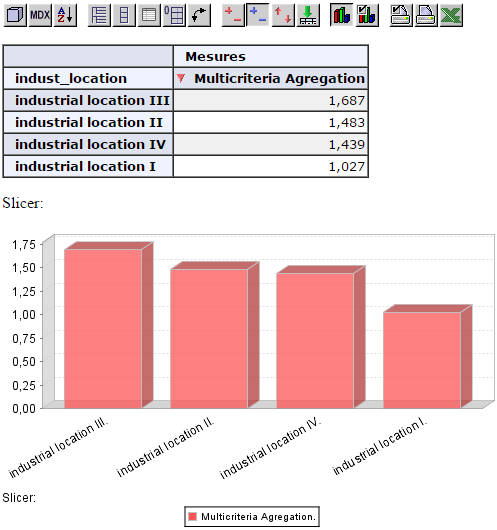


Combination 6


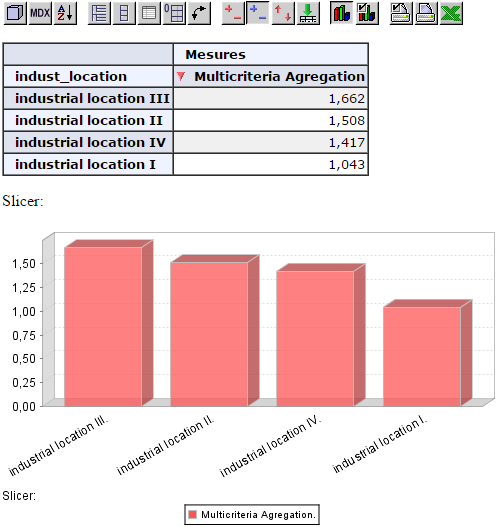


Combination 7


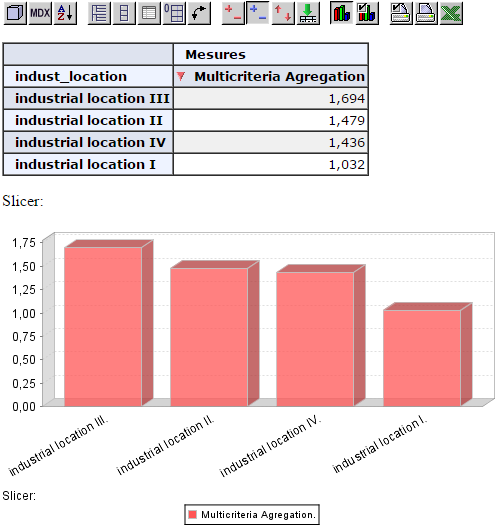


Combination 8


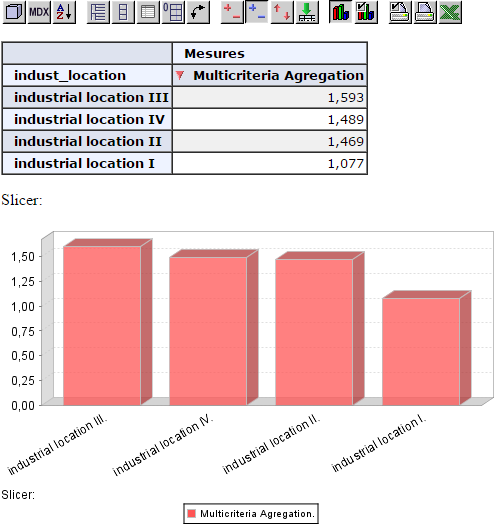


Combination 9


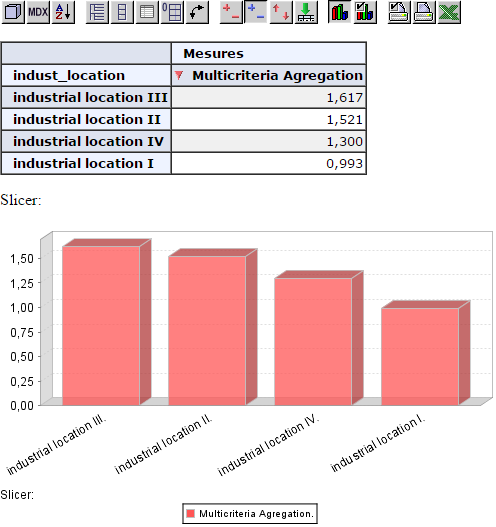


Combination 10


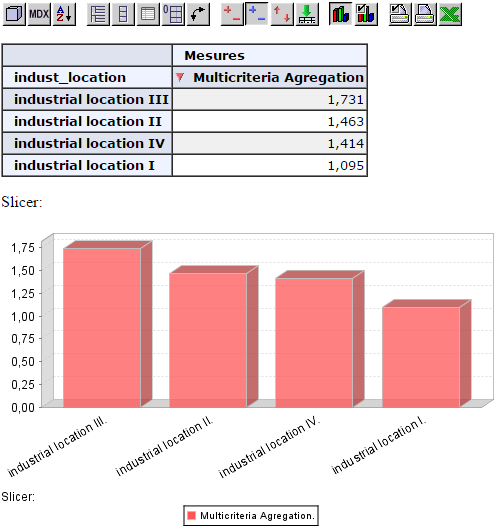


Combination 11


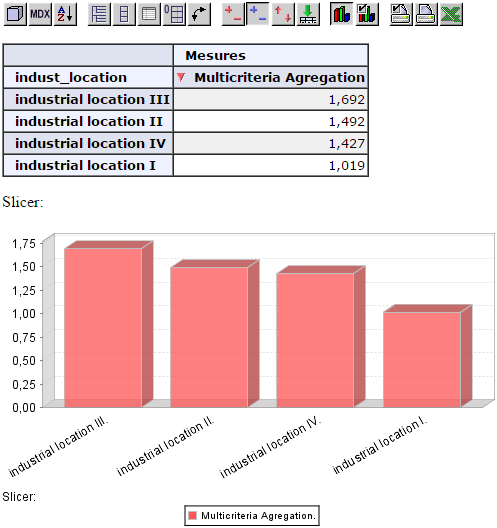


Combination 12


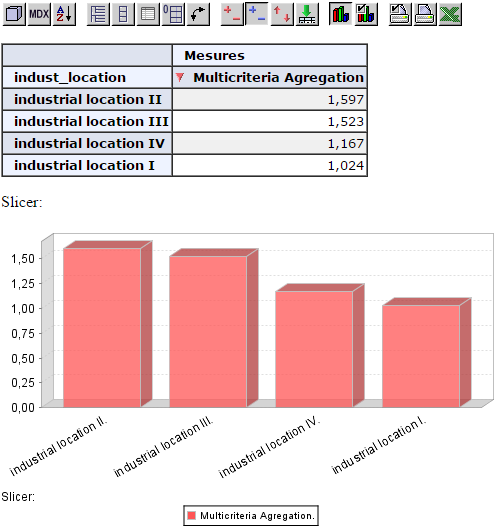


Combination 13


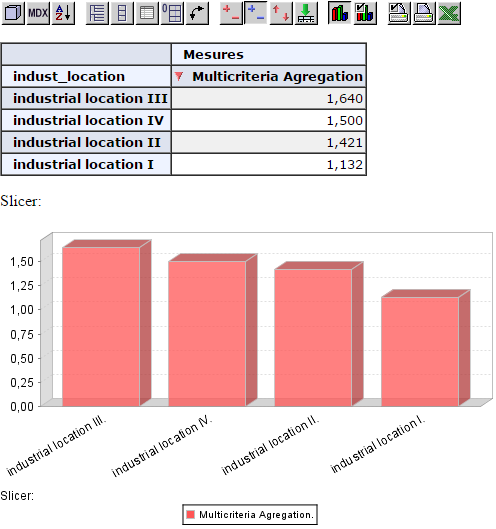


Combination 14


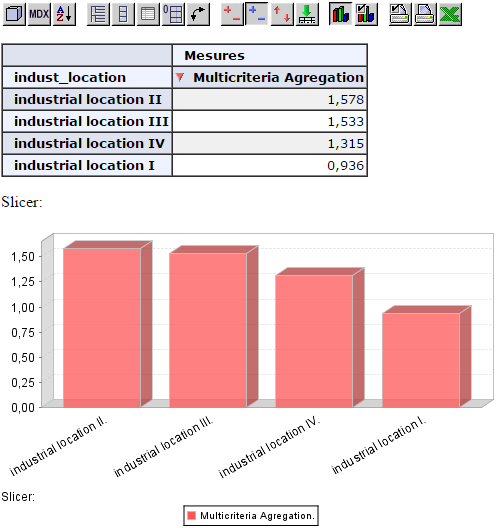


Combination 15


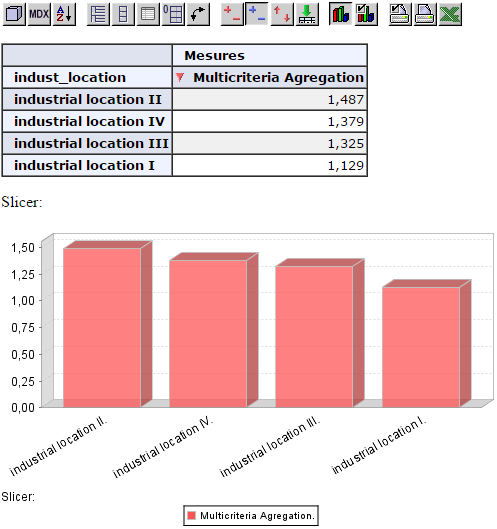

Supplement: Supplementary file 1 — 10.1186/s40064-015-1404-x The computational steps of fuzzy AHP process. [file 40064_2015_1404_MOESM1_ESM.docx]
